# Duropathies: A Narrative Overview of a Neglected Concept—Part One: Anatomical, Embryological, and Pathophysiological Elements

**DOI:** 10.3390/neurosci6040115

**Published:** 2025-11-14

**Authors:** Marialuisa Zedde, Rosario Pascarella

**Affiliations:** 1 Neurology Unit, Stroke Unit, Azienda Unità Sanitaria L ocale-IRCCS di Reggio Emilia, Viale Risorgimento 80, 42123 Reggio Emilia, Italy; 2 Neuroradiology Unit, Ospedale Santa Maria della Misericordia, AULSS 5 Polesana, 45100 Rovigo, Italy; rosario.pascarella@aulss5.veneto.it

**Keywords:** dural, pachymeninges, superficial siderosis, spontaneous intracranial hypotension, dural tear, CSF leak, ventral spinal fluid collection, spinal cord herniation, spinal arachnoid web, CT myelography, MRI

## Abstract

Duropathies encompass a spectrum of disorders linked to spinal dural tears and cerebrospinal fluid (CSF) leaks, resulting in significant neurological manifestations. This review synthesizes the current literature on duropathies, focusing on their anatomical and pathophysiological aspects, including conditions such as superficial siderosis, spontaneous intracranial hypotension, and spinal cord herniation. The methodologies employed include comprehensive evaluations through neuroimaging techniques such as MRI and CT myelography, alongside clinical assessments of symptoms like ataxia, hearing loss, and cognitive impairment. Key findings highlight the prevalence of dural defects in patients with superficial siderosis and the association of persistent CSF leaks with various neurological impairments. The review emphasizes the need for a standardized diagnostic and therapeutic approach to enhance patient management and improve outcomes. By addressing the interrelated nature of these conditions, the study underscores the importance of early intervention to mitigate long-term neurological consequences. Overall, the findings advocate for further research to elucidate the mechanisms underlying duropathies and the development of effective treatment strategies, ultimately aiming to improve the quality of life for affected individuals.

## 1. Introduction

The term “duropathies” was first introduced by Kumar et al. [1] to describe a group of diseases that share a common pathogenetic trigger: a spinal dural tear accompanied by a cerebrospinal fluid (CSF) leak within the spinal dura. The range of clinical disorders linked to dural defects includes craniospinal hypovolemia (often marked by orthostatic headaches), ataxia, hearing impairment associated with superficial siderosis (SS), segmental weakness and atrophy—either with or without hyperreflexia—and spinal cord herniation [2]. The clinical presentations of these disorders may overlap. Dural defect-related ventral longitudinal intraspinal fluid collection (VLISFC) can be found in all these conditions [3].

Duropathies can arise from multiple causes, such as traumatic injuries, congenital anomalies, and degenerative diseases. Spontaneous intracranial hypotension (SIH), which is characterized by low CSF volume due to dural tears, often manifests with orthostatic headaches and neurological deficits, underscoring the essential role of the meninges in sustaining CSF homeostasis [1]. Superficial siderosis, resulting from chronic bleeding into the subarachnoid space, can cause hemosiderin deposition and progressive neurological decline, particularly affecting the cerebellum and auditory pathways. Another significant manifestation of dural disease is spinal cord herniation, which occurs when the spinal cord protrudes through a dural defect, frequently resulting in debilitating symptoms such as weakness and sensory disturbances. Arachnoid webs—thickened arachnoid tissue that can compress the spinal cord—further complicate the clinical scenario by obstructing normal CSF flow and contributing to conditions like syringomyelia.

A thorough understanding of the underlying mechanisms and clinical implications of these duropathies is crucial for developing effective diagnostic and therapeutic approaches. This review consolidates the current literature on these conditions, aiming to provide a comprehensive overview of the anatomical, embryological, and pathophysiological factors that contribute to duropathies while highlighting the significance of prompt intervention to reduce long-term neurological consequences. This review has a narrative design and it is intended to be followed by a second review addressing the clinical, neuroradiological, and therapeutic issues of duropathies in detail. This first review seeks to explore the anatomical and pathophysiological aspects of duropathies to enhance understanding of the clinical and neuroimaging features, thereby establishing a cohesive perspective.

## 2. Anatomical and Pathophysiological Background

The organization of the meningeal layers is almost the same in all adult mammals, i.e., in three contiguous layers, maned from the outermost to the innermost, dura, arachnoid, and pia mater. Each layer maintains unique histological features, underlying their different functions and embryology. The main topic of this review is the dura mater, also called pachymeninx due to the thick collagenous content securely adhering to the inner surface of the skull (including the cerebellar tentorium and the cerebral falx). It includes two layers: (1) the outer endosteal (or periosteal) layer, constituting the periosteum for the internal surface of the skull; (2) the inner meningeal layer (or true dura mater), only separated by the previous one along the course of the dural venous sinuses and merged in the other locations. As previously introduced, the dura mater constitutes rigid reflected folds protruding into the cranial cavity, the falx cerebri, and the tentorium cerebelli [4]. Notably, the dura mater, especially its endosteal layer, is abundant in blood vessels that primarily supply the calvaria [4]. Furthermore, lymphatic vessels within the dura mater are responsible for draining cerebrospinal fluid (CSF) from the central nervous system (CNS) [5,6,7].

### 2.1. Embryology

The development of the meninges has been widely investigated across several species, including humans, but most experimental studies were conducted on mice and rats [5,8,9]. The process begins when the primitive meninx appears as connective structure interposed between the neuraxis and the vertebral canal. A currently unresolved point is if the primitive meninx gradually differentiates into the leptomeninx and the pachymeninx versus a separate origin of the pachymeninx from a distinct mesenchymal source [10]. Six stages have been identified in the development of the meninges in several species:

- During early embryonic stages, mesenchymal cells proliferate around the hindbrain as the neural tube closes, and after they extend to the midbrain and forebrain [11].
- By Carnegie stage 15 in humans (around the fifth gestational week), corresponding to embryonic day 9.5 in mice, the primary meninx appears from the organization in layer of mesenchymal cells. This is the precursor for the meninges, skull, and scalp [11]. This primary meninx is supported by a vascular network that later gives origin to the blood vessels supplying both the meninges and the brain [8].
- In the following stage, the presence of cells within the vascular plexus signals the differentiation of the pia mater, where fibroblasts produce extracellular matrix proteins that create a basement membrane, effectively separating the meninges from the brain [11].
- The next step is around embryonic day 10.5 in mice, when the primary meninx splits into an outer dense layer and an inner reticular layer. This last one is probably the main contributor to the formation of the three meningeal layers.
- In stage 17 in humans (the sixth gestational week), corresponding to embryonic day 13 in mice, the mesenchyme above the primitive meninx differentiates into layers, specifically the dermal and the “calvarial” ones, which gives rise to the skull and its sutures [11,12,13].
- The primitive meninx finally differentiates into the pachymeninx (composed of longitudinally arranged fibroblasts) and leptomeninx. The dural limiting layer, a sheet of packed cells, may contribute to the dura and arachnoid mater, explaining the inclusion of the outer arachnoid in the definition of pachymeninx [8].
- Moreover, the differentiation of the meningeal layers occurs in the basal to the apical direction [14]. The leptomeninx becomes cavitated, giving origin to the arachnoid trabeculae and the subarachnoid space, while the dura mater becomes thicker, accumulating collagen fibers [15].

Additionally, lymphatic vessels within the dura mater start to develop during the early postnatal period [16]. The spinal and telencephalic meninges have markedly different origins. The process of meningeal development is intricate, and its precise origins in mammals are still not fully understood. Early research conducted by Harvey and Burr on amphibian embryos proposed that the cranial dura mater is derived from mesoderm, whereas the arachnoid and pia mater are believed to stem from the neural crest [17,18]. Later investigations utilizing quail-chick chimeras revealed that the meninges associated with the telencephalon originate from the mesencephalic neural crest [19]. Conversely, research by Bagnall et al. [20] and Halata et al. [21], utilizing similar chimera models, proposed that all layers of the meninges have a mesodermal origin, reinforcing earlier findings by Le Lièvre [22] and Le Douarin [19] showing that the neural crest does not contribute to the meninges of the spinal cord. At present, it is not possible to experimentally track the origins of the meninges in mammals; however, the findings indicate similarities with avian developmental processes [23]. Histological analyses of human embryos show that the neural crest plays a role in the formation of the pia mater, whereas the paraxial somitic mesoderm is involved in the development of the dura mater [11].

The development of the meninges in rat fetuses closely resembles various stages of phylogenesis. In rats, which have a developmental period of 21 to 23 days (D), the meninx primitiva is formed by D12. By D14, a reticular network begins to appear, paving the way for the formation of the subarachnoid space, and by D15, the ectomeninx differentiates from the endomeninx. The pia mater, which arises from the arachnoid mater, differentiates later, around D20. Although the differentiation of the meningeal layers is nearly concluded before birth, the proliferation of arachnoid cells persists into the early neonatal period [15,24]. In humans, the differentiation of the three meningeal layers is completed by the third month of intrauterine life. During the early stages of development, the central nervous system is closely associated with the mesodermal sheath formed by the meninx primitiva, which is clearly defined by day 33 (D33). The dura mater begins to differentiate on the ventral side of the spinal cord around day 44 (D44) and extends dorsally to the spinal ganglia by day 52 (D52), while the pial layer subsequently differentiates on day 57 (D57). The formation of the pia–arachnoid interface coincides with the differentiation of the ependymal lining of the ventricles. In the early fetal stages (approximately 30 mm in length, 56–60 days of gestation), vertebral growth outpaces spinal cord growth, resulting in the division of the meninx primitiva into external and internal layers. The external layer adheres to the bony envelope and develops into the ectomeninx, which will become the spinal dura mater. The internal layer, known as the endomeninx, will give rise to the pia mater, the arachnoid layer, and the arachnoid reticulum. The epidural cavity begins to form starting from day 57 (D57). The subarachnoid space develops between the trabeculae of the arachnoid reticulum. Histological studies of human embryos at various developmental stages, supported by experimental findings, indicate that the formation of the subarachnoid space is intrinsic to the meningeal tissue and not merely a result of mechanical effects from CSF flow. Notably, the subarachnoid space first appears on the 32nd day at the ventral side of the rhombencephalon, while the fourth ventricle remains closed. Additionally, CSF circulation does not commence until the 41st day, which is nine days after the initiation of subarachnoid space formation. The underlying mechanisms of this process are not yet fully understood, but capillaries may play a crucial role in CSF secretion and absorption during embryogenesis, as indicated by the presence of intracranial arachnoid cysts along arterial supplies.

As human development progresses, structures associated with the tail undergo a gradual regression. By the 11th week of gestation, the spinal cord, encased in its meningeal sheath, contracts into the filum terminale and the coccygeal ligament. The filum terminale is essentially a pial sheath that surrounds neural structures formed as the spinal cord regresses. Up until the early part of the third month of gestation, the spinal cord occupies the entire vertebral canal and extends down into the coccygeal area. However, as development continues, the growth of the vertebral column and the meninges begins to surpass that of the spinal cord. By the end of the fifth month, the spinal cord terminates at the base of the sacrum. At full term, the spinal cord measures approximately 15 to 17 cm, with the conus medullaris positioned at the level of the third lumbar vertebra. This significant upward migration of the spinal cord leads to the lower segment of the vertebral canal, below the third lumbar vertebra, being filled with lumbosacral nerves, the filum terminale, and blood vessels, all enveloped in their respective meningeal sheaths. In addition, the epidural space outside the dural sac contains epidural fat, blood vessels, and the filum of the spinal dura mater. The details of the regulation of the meningeal development are summarized in Table 1.

In addition, the development of the meninges regulates the development of the calvarium (Table 2).

The meninges play an essential role in the development of the brain, carrying out a variety of functions that change over time. At first, they supply vital trophic factors that are crucial to the survival of brain cells. Research involving the removal of the neural crest in early chick embryos, which hinders the formation of forebrain meninges, has demonstrated considerable apoptosis and degeneration of the neuroepithelium in the forebrain, although the specific factors involved have not yet been identified [54]. Additionally, the meninges are involved in regulating neuronal migration by releasing molecules that can attract or repel cells. They generate a chemoattractant known as CXCL12 (SDF-1), which is activated by FOXC1 and helps guide neurons and neural progenitors expressing CXCR4 and CXCR7 towards the marginal zone located beneath the meninges [55,56,57]. On the other hand, factors such as BMP4, BMP7, and TGFβ1 cause oligodendrocyte precursor cells to move away from the ventral forebrain towards the cerebral cortex [58]. Moreover, signaling from RA influences the migration of cortical neurons; the deletion of an enzyme responsible for synthesizing RA in the meninges disrupts neuronal migration and the organization of cortical layers [59]. The pial basement membrane serves a structural purpose in directing neuron migration. Radial glial cells extend their processes from the ventricular zone to the surface of the brain, creating a scaffold for neurons on the move, with their endfeet anchored to the pial basement membrane [8,60,61]. In mouse mutants that exhibit defects in this membrane, the early detachment of radial endfeet results in an abnormal distribution of neurons within the cerebral cortex and cerebellum [37,59,62,63].

Additionally, the meninges have a significant effect on neurogenesis by modulating asymmetric cell divisions. In Foxc1 mutants that lack most of the meninges, researchers observed an increase in symmetric divisions coupled with a decrease in asymmetric divisions, leading to an elongated cerebral cortex with a reduced number of differentiated neurons. Interestingly, treating the embryos with retinoic acid (RA) in utero partially mitigated the cortical abnormalities, highlighting the role of meningeal-derived RA in cortical neurogenesis [60]. BMP7 is also crucial for neurogenesis within the embryonic dentate gyrus [64]. Furthermore, the meninges are integral to the formation of brain blood vessels. In Foxc1ch/ch and Foxc1lacZ/lacZ mutants, blood vessels in the cerebral cortex exhibited larger diameters but decreased density due to the absence of meninges and a loss of RA [30]. RA signaling influences the WNT/β-catenin pathway in endothelial cells, facilitating normal vascular development and inhibiting improper growth [65]. Recent research has indicated that the synthesis of BMP2 and BMP4, which is regulated by TWIST1 in the dura mater and calvarial bone, is essential for the growth and remodeling of cerebral veins [33]. Furthermore, the meninges play a role in the development of the corpus callosum; BMP7 inhibits the outgrowth of callosal axons while WNT3 from neurons counteracts this inhibition, promoting the formation of the corpus callosum [35]. The meninges also establish a niche for neural stem cells, with a specific population expressing Nestin identified in the leptomeninges, acting as stem cells for cortical neurons [66,67,68]. Due to their vital role in brain development, abnormalities in the meninges have been associated with neurodevelopmental disorders.

Cobblestone lissencephaly, which is marked by the absence of typical ridges in the cerebral cortex, results from compromised integrity of the pial basement membrane. This leads to excessive neuronal migration into the meningeal layers and is often associated with mutations in genes related to the extracellular matrix [69,70]. Dandy–Walker malformation, characterized by cerebellar hypoplasia and hydrocephalus, has been linked to the FOXC1 gene, which is expressed in the meninges but not in the brain itself. This suggests that abnormalities in the meninges may contribute to the development of this condition [71,72]. Imaging studies on individuals with FOXC1 mutations have revealed signs of meningeal deficiency, although the precise meningeal phenotype remains unclear for many patients [29,72].

### 2.2. The Cranial Dura Mater

The dura mater, which is the outermost layer among the three meningeal membranes, is distinguished by its thickness, density, and lack of elasticity, effectively encapsulating the brain and spinal cord while preventing the leakage of CSF. This layer is rich in fibroblasts, contributing to its flexibility [4]. It has a shared embryological origin with the fascial system. Anatomically, the dura mater varies between the cranial and spinal regions, yet they form a continuous membrane at the foramen magnum. The dural system is essential for supporting brain functions in conjunction with CSF, serving as a protective barrier against mechanical injuries. Mechanical forces applied to the skull are dampened through shock absorption, supported by the elasticity of bone, the structural arrangement of skull pillars, nasal sinuses, and the connection between the viscerocranium and neurocranium [4].

Inside the cranial cavity, the dura mater lines the inner surface of the skull and is composed of two distinct layers: the outer endosteal layer and the inner meningeal layer. These layers are typically in close contact, but separations can occur in areas where venous sinuses develop to aid in blood circulation. The cranial dura is made of white fibrous tissue and includes a specialized layer of flattened fibroblastic cells that lack extracellular space and collagen, situated between the dura mater and the arachnoid layer. The outer surface of the dura adheres firmly to the inner surfaces of the skull bones, with particularly strong connections at the base of the skull and areas away from the sutures. Its outer surface is rough and fibrillated, while the inner surface is smooth and lined with endothelium. The dura mater extends to the outer surface of the skull through specific foramina at its base, forming fibrous sheaths for the nerves as they exit the cranium through these openings. At the foramen magnum, the dura mater merges with the bone, transitioning into the spinal dura mater. Emissary veins link the scalp skin to the dura, and the dura appears particularly flat in regions associated with the ethmoid bone cells, the tegmentum, and the sigmoid sinuses. The inner meningeal layer of the dura is structurally less robust than the outer endosteal layer [73]. Research suggests that the dura in adults can endure greater forces compared to neonates [74]. Arbuckle proposes that a specific arrangement of fibers enables the cranial and spinal dura to transmit different mechanical forces [75]. These “stress fibers” are organized into horizontal, vertical, transverse, and circular patterns. This unique arrangement in the cranial dura mater likely develops in response to mechanical forces experienced during embryonic development, allowing collagen fibers to align according to stress [76].

### 2.3. The Spinal Dura Mater

The spinal dura mater (SDM) is a rigid tube composed of collagen fibers that loosely encases the spinal cord, maintaining tension influenced by cerebrospinal fluid (CSF) and various forces, including hydrostatic, respiratory, and pulsatile pressures [77]. It extends from the occipital bone at the foramen magnum down to the sacral canal, where it merges with the filum terminale at the S3 level. A subdural space, which typically contains a small volume of CSF, separates the SDM from the arachnoid mater. Structurally, the SDM consists of three layers: an outer fibroelastic layer, a middle fibrous layer, and an inner boundary cell layer characterized by extracellular spaces and limited cell junctions. Elastic fibers within the dura contribute to its flexibility. The extradural space plays a role in distributing the forces acting on the spinal cord and meninges. The outer periosteal layer of the dura terminates at the foramen magnum, while the inner layer represents the true spinal dura mater.

Unique to the spinal region, the epidural space allows for gliding movements between the dura and the spinal column. Anteriorly, this space is bordered by the posterior longitudinal ligament, and at the foramen magnum, the dura integrates with the periosteum. The dura mater is notably thicker in the cervical and lumbar regions compared to the thoracic region. Additionally, the cranial durae matris spinalis (CDMS ligament) consists of fibrous strands connecting the dura mater to various anatomical structures, providing tension mechanisms for the upper cervical vertebral column during movement [78,79,80]. Connections between the SDM and surrounding muscles, such as the rectus capitis posterior minor (RCPmi), prevent folding of the SDM during neck extension. The myodural bridge is clinically significant, as stresses transmitted via these connections can lead to cervicogenic headaches [81,82,83,84]. Various attachments, including connections to the nuchal ligament and the flaval ligaments, further stabilize the SDM and spinal cord [79,85,86,87,88,89,90,91,92]. Embryologically, the spinal dura mater originates from somitic mesoderm, while the arachnoid and pia mater derive from the neural crest [93]. The meninx primitiva gives rise to the dura mater, forming between the arachnoid mater and the calvarial mesenchyme, with initial structures like dural reflections developing first [94]. Studies involving amphibian embryos and quail-chick chimeras support the idea that the cranial dura mater originates from mesoderm, while telencephalon meninges arise from the mesencephalic neural crest [11,12,13]. In rats, the meninx primitiva is evident by embryonic day 12 (D12), with the subarachnoid space starting to form by D14 and the ectomeninx differentiating by D15. In humans, the three meningeal layers complete differentiation by the third month of intrauterine life, with the dura mater beginning to differentiate around D44 [95]. The pia–arachnoid interface forms concurrently with the differentiation of the ependymal lining [20,21,22]. Development of the subarachnoid space occurs independently of mechanical actions from CSF flow [96].

In the cranial region, the spinal dura mater begins at the foramen magnum and gradually narrows into the filum terminale at the S1 segment. It firmly attaches to the periosteum of the spinal canal and surrounds both the dorsal and ventral nerve roots in tubular sheaths, becoming progressively thinner as it approaches the spinal ganglia [97]. Surgical interventions at the foramen magnum necessitate meticulous dissection of the dura mater. This layer is anchored to the dorsal longitudinal ligament through strong attachments to the vertebrae, particularly in the cervical and lumbar regions, which provide stability to the spinal cord during movement [98,99,100]. Moreover, the spinal dura mater has a relatively low vascular supply, primarily obtaining blood from radicular arteries that branch off larger arteries, leading to slender spinal dural arteries (Table 3) [101].

The arterial supply of the spinal dura mater is structured in a metameric fashion, influenced by various anastomoses. The ventral portion of the spinal dura has limited vascularization from a median source, while the dorsal portion is well-supplied by two dorsolateral sources, mirroring the arterial supply of the spinal cord beneath. Spiral arteries and capillary networks are present on the outer surface of the dural sheath, particularly protruding into the epidural space on the dorsal side of the thoracic region. However, the functional role of these structures remains uncertain, and there is ongoing debate regarding the organization of the arterial network [101].

The physiology of the spinal dural venous network and its relationship with spinal cord drainage is not completely understood. Traditional anatomical descriptions disagree with reports from interventional neuroradiologists. Anatomical research reveals a network of perimedullary veins that drain into both ventral and dorsal radicular veins, which pass through the dura mater and exit the vertebral canal via intervertebral foramina. These radicular veins are equipped with valves to prevent retrograde blood flow into the intradural venous network, thereby shielding the spinal cord from increased blood pressure in the vertebral venous system during activities such as physical exertion or coughing. Generally, two veins accompany each dural artery, draining into the anterior and posterior internal vertebral plexuses located in the epidural space. At the cervical level, these veins form sizable venous sinuses that connect with the basilar plexus to facilitate cranial venous drainage. However, interventional neuroradiologists have observed that cranial venous drainage can occur through perimedullary veins without any communication with epidural veins, especially when the patient is in a supine position [44].

The anatomy of lymphatic drainage within the spinal meninges continues to be a subject of considerable debate among researchers and anatomists. Early investigations that involved the injection of ink into the ventricular or subarachnoid spaces revealed notable accumulation around the cervical and lumbosacral nerve roots. This observation led to the hypothesis that lymphatic vessels could potentially originate from the dura mater, particularly at the sites where the denticulate ligaments attach and at the lumbar vertebral bodies [102].

Furthermore, it has been suggested that fine lymphatic vessels are responsible for draining the ventral aspect of the meningeal recesses associated with the nerve roots, directing this lymphatic fluid into nearby paravertebral lymph nodes. However, it is important to note that the specific locations of drainage are not yet well established and remain somewhat ambiguous in the existing literature. In the thoracic region, lymphatic vessels typically drain into the posterior mediastinal lymph nodes, while those associated with the lumbosacral area direct their flow towards lymph nodes located in the posterior abdominal wall [103,104]. Additionally, the movement of chemical markers into the lymphatic pathway following injections has provided evidence supporting the involvement of these lymphatic vessels in the absorption of CSF [105]. This lymphatic pathway may serve as an alternative route for the absorption of CSF, which could be particularly significant in certain populations, such as neonates and elderly individuals, where the functionality of the arachnoid villi, which are normally responsible for CSF absorption, may be compromised [10].

Despite these insights, the exact physiological role of this lymphatic drainage pathway remains largely unclear. Nevertheless, it is suggested that spinal CSF outflow could play a crucial role in human physiology, especially during activities that require an upright posture or during instances of physical exercise [106]. This aspect underscores the complexity of fluid dynamics within the spinal system and highlights the importance of further research to elucidate the intricacies of lymphatic drainage in the spinal meninges.

The inner surface of the spinal dura mater is lined by the outer arachnoid layer, which extends laterally to accommodate the nerve roots that pass through the dura mater at the intervertebral foramina. Between these foramina, the dura mater connects with pial structures known as the ligamenta denticulata, which are located beneath the deeper layer of the arachnoid mater. At each spinal segment, the ventral and dorsal nerve roots penetrate the dura through distinct openings, each encased in individual dural sheaths before converging to form a spinal nerve, which is enveloped by a single dural sheath. The first cervical nerve roots are positioned adjacent to the vertebral artery as they navigate through the dural sheath. Within the intervertebral foramen, the spinal nerve and its accompanying radicular artery are centrally located, with the radicular artery typically lying ventrally to the spinal nerve, often situated outside of the dural sheath. Surrounding these vessels is the intervertebral venous plexus, with branches extending toward the dorsal ganglion to create the periganglionic plexus. The connective tissue encasing these vessels merges with the meninges as they traverse the dura. Additionally, the sinuvertebral nerve runs ventrally to the spinal nerve, located externally to the dural sheath. The intervertebral foramen is laterally sealed by a fibrous operculum, with the dural sheath of the spinal nerve covering the inner aspect of this operculum and fusing with the periosteum of the vertebra. Laterally, the dural sheath transitions seamlessly into the epineurium of the nerve trunk, forming the lymphatic epidural space where medical injections can be administered [107]. This intricate arrangement highlights the complex interactions between the various structures involved in spinal anatomy and their functional significance in both health and medical procedures.

### 2.4. The Role of Spinal Leptomeniges

The arachnoid mater is a delicate and transparent membrane that envelops the spinal cord, spinal nerve roots, cauda equina, and the associated spinal vessels. Its outer layer is anchored to the dura mater by fine strands of collagen, which effectively prevents the formation of a subdural space. This outer layer is connected to the pia mater through trabecular structures and the dorsal septum of Schwalbe. The subarachnoid space, which is a crucial component of the central nervous system, occupies approximately one-third of the lumen of the vertebral canal. It connects with the cranial subarachnoid space at the foramen magnum and expands into a terminal sinus that encases the cauda equina, concluding between the first and second sacral vertebrae. Within this space, the subarachnoid area is divided into ventral and dorsal chambers by the ligamenta denticulata, with trabeculae extending and pressing against the surface of the spinal cord. This arrangement plays a significant role in supporting the spinal cord and facilitating the flow of CSF, which is essential for cushioning and nourishing the neural structures. Overall, the arachnoid mater’s intricate architecture underscores its vital function in maintaining the health and stability of the spinal cord and its associated elements [108,109].

Lateral recesses create CSF sleeves around the spinal nerve roots, formed by the fusion of two layers of arachnoid mater. This region is notable for containing cellular debris and activated macrophages, suggesting that it plays a significant role in the immune defense mechanisms of the central nervous system and the CSF space [110,111]. These recesses can be effectively visualized using diagnostic imaging techniques such as Myeloscan and T2-weighted MRI, which are particularly important for evaluating conditions like disk herniation and vertebral canal stenosis. The anatomical characteristics of these lateral recesses differ across various vertebral segments. Specifically, the subarachnoid sleeves tend to be shorter in the thoracic region compared to those in the cervical and lumbar areas. Understanding the anatomical nuances of these recesses is essential, especially for clinicians performing image-guided corticosteroid infiltrations. The precise knowledge of their structure and location can greatly enhance the accuracy and effectiveness of such interventions, ultimately contributing to improved patient outcomes in managing spinal conditions.

The arachnoid mater is unique in that it does not possess its own specific vascular supply; instead, it depends on the blood vessels of the spinal cord for its nourishment. Within the subarachnoid space, CSF is secreted primarily by the choroid plexuses. For clinical purposes, lumbar punctures are typically performed between the third and fourth lumbar vertebrae to safely collect CSF samples. This fluid serves a critical function by providing hydromechanical protection to the spinal cord, cushioning it against potential injury. Interestingly, the arachnoid mater has the capacity to form proliferations that resemble the cranial arachnoid granulations, which are known to be involved in the reabsorption of CSF [112]. Researchers have identified three distinct types of spinal arachnoid proliferations based on their anatomical relationship with the dura mater and the surrounding epidural veins [113]. These proliferations may contribute to approximately 25% of CSF drainage in various animal models [102,114,115]. In humans, spinal arachnoid villi might also play a significant role in the absorption of CSF, particularly under specific physiological conditions [106]. This highlights the arachnoid mater’s adaptability and potential contributions to maintaining fluid balance within the central nervous system.

The spinal pia mater, often referred to as the vascular meninx, is composed of thin areolar tissue that closely adheres to the glia limitans of the spinal cord. This membranous layer extends cranially, transitioning into the cranial pia mater at the foramen magnum, and continues downward to form a tubular structure known as the filum terminale, which is located beneath the conus terminalis. At the interface between the spinal dural sheath and the spinal ganglion, the pia mater fuses with the arachnoid layer, giving rise to the perineurium. In comparison to the cranial pia mater, the spinal pia is thicker and exhibits a lower degree of vascularization. A collagenous subpial layer acts as a barrier, separating the pia mater from the glia limitans [109]. Advanced imaging techniques, such as scanning electron microscopy, have shown that the pia mater forms a continuous, non-fenestrated layer, effectively isolating the CSF from the extracellular space of the spinal cord.

Within the subarachnoid space, blood vessels are enveloped by a leptomeningeal layer that is continuous with the pia mater. This layer surrounds the small vessels that penetrate into the spinal cord. Among the notable structures associated with the pia mater are the linea splendens, which is a thickening of the pia on the ventral side, and the ligamenta denticulata, which serve to stabilize the spinal cord within the subarachnoid space. The vascularization of the pia mater is supplied by the blood vessels of the spinal cord, which are fewer in number compared to those found in the cranial pia mater. Additionally, the pia mater is innervated by a loose plexus known as the plexus of Purkinje, which is formed by vasomotor and sensory nerves that originate from the surrounding vascular plexuses [109]. This intricate arrangement underscores the vital role of the spinal pia mater in both protecting the spinal cord and facilitating its vascular needs.

### 2.5. The Epidural and Subdural Space

The epidural space is situated between the dura mater and the osteofibrous wall of the vertebral canal, defined ventrally by the dorsal longitudinal ligament and the dorsal aspect of the vertebral bodies and intervertebral disks. Dorsally, it is bounded by the spinal laminae and ligamenta flava, while caudally, it is enclosed by the sacral hiatus, which is closed by the coccygeal ligament. Cranially, the space extends from the dura mater at the foramen magnum to the posterior aspect of the third vertebral body. Notably, there is no intracranial epidural space, and the lateral limits are formed by the vertebral pedicles and intervertebral foramina, which are closed by fibrous opercula covered with the dural sheath of spinal nerves. The dimensions of the epidural space vary by vertebral segment, being maximal at the lumbar segment (5 to 6 mm) and thinning in the cervical region.

The epidural space, situated between the bony vertebral canal and the dura mater, is characterized by the presence of loose connective tissue and epidural fat. The amount of fat within this space can vary significantly depending on an individual’s overall body fat distribution. Notably, the ventral epidural space contains minimal amounts of epidural fat, which can complicate procedures such as catheter placement for anesthesia, often resulting in coiling of the catheter. Within the epidural space lies the internal vertebral venous plexus, which consists of both ventral and dorsal plexuses that extend from the foramen magnum all the way down to the coccyx. These plexuses are interconnected by transverse anastomoses and are unique in that they lack valves, positioning themselves on either side of the dorsal longitudinal ligament. At the cervical level, the epidural veins establish lateral connections with the vertebral plexus. In both the cervical and lumbar regions, these veins are generally positioned along the ventrolateral sides of the vertebral canal, with a notable lack of a posterior epidural vein. Superiorly, the cervical epidural veins connect to the suboccipital plexus as well as the anterior condylar veins. These veins act as emissary veins, establishing a link between the suboccipital plexus and the lateral sinuses. This venous network is crucial for draining blood from the brain, particularly in scenarios such as maintaining an upright posture or during episodes of intracranial hypotension, when the jugular veins may become compressed. In these circumstances, the cervical internal plexus plays a vital role in facilitating venous outflow from the intracranial space [116]. This intricate network underscores the significance of the epidural space in maintaining proper venous drainage and supporting overall cerebrospinal dynamics. The internal vertebral plexus plays a crucial role in venous drainage, receiving tributaries from both the vertebrae and the spinal cord. It establishes communications with external plexuses and ultimately drains into the superior and inferior vena cava. Specifically, the cervical internal vertebral plexus connects with the suboccipital sinus, facilitating drainage into the vertebral vein, internal jugular vein, and deep cervical veins, which all converge to lead into the superior vena cava. In the thoracic region, the epidural plexus drains into the hemiazygos veins, while the lumbar plexus provides connections to both the superior and inferior vena cava. This epidural venous system is characterized as a valveless anastomotic network, which is influenced by gravitational forces as well as factors such as body posture and the pressures exerted by the thoracic and abdominal cavities. Interestingly, valves present in the radiculomedullary veins serve a protective function for the spinal cord, helping to prevent venous congestion during periods of elevated venous pressure.

Surgical interventions in the thoracolumbar spine can become complicated due to the potential for increased venous pressure, particularly when abdominal wall pressure is applied against the operating table. Additionally, within the epidural space, the cauda equina nerve roots are housed within their meningeal sheath, providing a critical pathway for therapeutic interventions. This space is utilized for anti-inflammatory treatments, with glucocorticoids being injected to alleviate conditions such as sciatica and lumbar stenosis. This multifaceted role of the epidural space highlights its importance not only in venous drainage but also in the management of various spinal conditions [117,118,119].

The subdural space, positioned between the dura mater and the arachnoid layer, is often regarded as an artifact, typically arising from surgical manipulation or histological preparations. This space is characterized by thin trabeculae that connect the dura and arachnoid layers. Advanced imaging techniques, such as transmission electron microscopy, have revealed a dura-arachnoid interface populated by neurothelial cells, which facilitate the safe opening of the dura without compromising the integrity of the arachnoid layer. Under normal physiological conditions, the dura mater remains closely attached to the arachnoid, indicating that the subdural space should not be viewed as a true “virtual” space, akin to the pleural cavity. Instead, it represents a potential space that can become evident under certain conditions, such as trauma or disease. In clinical practice, it is essential to differentiate between various types of cystic lesions, including meningeal cysts, perineurial cysts, and ganglion cysts, as they can often be confused due to their overlapping characteristics. Each of these lesions has distinct anatomical and clinical implications, making accurate diagnosis critical for effective management and treatment. Understanding these differences is vital for healthcare professionals when assessing spinal or neurological conditions.

Meningeal cysts can be extradural or intradural, with extradural cysts commonly found in adolescents, often associated with congenital defects in the radicular dura mater [120]. Intradural cysts have walls composed of arachnoid epithelium and communicate with the radicular subarachnoid space [121]. Perineurial cysts (Tarlov cysts) occur along sacral nerve roots and are filled with nerve fibers and ganglion cells, with no communication to the subarachnoid space [122]. Their pathophysiology is debated, but they may arise from congenital defects or inflammation around nerve roots [123]. Ganglion cysts in the lumbar epidural space are not connected to the meninges and result from degeneration of connective tissue near synovial joints [124].

## 3. Spinal Dura Mater and Its Functional Role

The meninges and their surrounding structures provide essential mechanical protection to the neuraxis. Unlike the cranial meninges, which are closely attached to the skull, the spinal meninges are positioned away from the bony walls of the vertebral canal, safeguarding the spinal cord from injuries caused by vertebral movements. Epidural fat, the internal vertebral venous plexus, and fluid in the subarachnoid space act as a hydromechanical cushion, buffering the spinal cord from surrounding bony structures. The dura mater is anchored to the foramen magnum, intervertebral bodies, and coccyx, ensuring stability during vertebral movements.

In adults, CSF is produced at a rate of approximately 400 to 600 mL per day, while the total volume of CSF in the system is about 150 to 160 mL. Of this volume, around 38.8 ± 11.4 mL is found in the lumbosacral subarachnoid space [125], and approximately 90 mL is located in the cranial subarachnoid space [126]. This distribution suggests that the volume of CSF surrounding the spinal cord itself may be quite minimal, potentially less than 5 mL. CSF circulates from its production sites in the choroid plexuses to various absorption sites, with a significant portion being absorbed by the spinal arachnoid villi [127]. Any unabsorbed CSF is returned to the cranial subarachnoid space. Research by Quencer et al. [128] has demonstrated that CSF circulates within the subarachnoid perimedullar space in opposing directions within the ventral and dorsal chambers, with the flow reversing direction with each heartbeat. This movement is not uniform; rather, it is pulsatile, influenced by the systolic pulse wave of the heart and respiratory movements [129]. This dynamic flow of CSF is vital for maintaining its roles in cushioning the central nervous system and facilitating metabolic exchange. CSF absorption mainly occurs through cranial arachnoid granulations, but additional absorption sites near spinal nerve roots have been identified.

Spinal arachnoid villi, which contact the epidural venous plexus, may play a crucial role in CSF absorption, especially during physical activity. Some arachnoid villi partially cross the dural sheath, providing surfaces for exchange based on the plication of the arachnoid layer. In green monkeys, approximately 16% of spinal roots exhibit arachnoid villi that penetrate the walls of veins surrounding the spinal ganglia [113]. This penetration plays a role in CSF absorption, which is modulated by CSF pressure; notably, the upright position may enhance this absorption process. Additionally, spinal lymphatic vessels are implicated in the CSF outflow system, suggesting an alternative pathway for the absorption of spinal CSF [130]. Research indicates that injected carbon particles can circumvent the venous system by traveling through arachnoid proliferations into the epidural space. This transport may be facilitated by lymphatic vessels, leading to the paravertebral lymph nodes [131]. Such findings highlight the complexity of CSF dynamics and the potential for lymphatic pathways to contribute to its regulation.

The subdural space, situated between the dura mater and the arachnoid layer, is often regarded as an artifact, typically resulting from surgical manipulation or histological preparations. Within this space, trabeculae connect the outer surface of the arachnoid to the inner surface of the dura mater, allowing for the safe opening of the dura without causing damage to the arachnoid. In clinical practice, it is important to differentiate between various cystic lesions, including meningeal cysts, perineurial cysts, and ganglion cysts, as these distinct entities can often be confused due to their similar presentations. Recognizing their differences is crucial for accurate diagnosis and appropriate management. Meningeal cysts can be either extradural or intradural; extradural cysts are typically found in adolescents and associated with congenital defects in the radicular dura mater [120]. Intradural cysts have walls made of arachnoid epithelium and communicate with the radicular subarachnoid space [121]. Perineurial cysts (Tarlov cysts) occur along sacral nerve roots, filled with nerve fibers and ganglion cells, and do not communicate with the subarachnoid space [122]. Their pathophysiology may involve degenerative processes or congenital defects [123]. Ganglion cysts in the lumbar epidural space are unrelated to the meninges and result from degeneration of connective tissue near synovial joints [124].

CSF turnover occurs approximately four to five times a day in young adults, although this rate tends to decrease with age. The pressure of CSF is determined by a dynamic equilibrium between its secretion, absorption, and the resistance to flow, with normal pressure ranges typically falling between 10 to 15 mm Hg in adults and 3 to 4 mm Hg in infants. Several factors can influence CSF pressure, including the systolic pulse wave, the respiratory cycle, abdominal pressure, and body posture. CSF plays a crucial role in maintaining homeostasis within the CNS. It is responsible for regulating electrolyte balance, facilitating the circulation of active molecules, and aiding in the elimination of metabolic waste products (catabolites). The circulation of CSF through the subarachnoid space is essential for modulating the activity of various functional regions of the CNS, supporting both protective and physiological functions vital for neural health and function. This intricate balance of factors and functions underscores the importance of CSF in maintaining the overall well-being of the CNS.

## 4. The Spectrum of Duropathies and Their Pathophysiology

Dural abnormalities are frequently observed in infratentorial superficial siderosis (SS), with dural tears identified as the primary cause in over 80% of affected patients, particularly those with spinal siderosis and ventral longitudinal intraspinal fluid collection (VLISFC) [125]. This association has given rise to the term “duropathies,” which encompasses conditions such as superficial siderosis of the central nervous system, SIH, multisegmental amyotrophy, and spinal cord herniation. Despite the identification of these overlapping duropathies, the underlying pathophysiology and interconnections remain poorly understood. The specific types of dural defects can provide insights into the symptoms presented by patients; however, it is still unclear why some individuals exhibit only a single manifestation of the duropathy spectrum, while others display multiple conditions. This variability in clinical presentation suggests a complex interplay of genetic, environmental, and possibly developmental factors that influence the severity and range of symptoms experienced by each patient.

Embryologically, the development of the meninges, including the dura mater, is crucial in understanding these conditions. The meninges arise from the primitive meninx during embryonic development, influenced by both mesodermal and neural crest cells. This differentiation process is essential for establishing the protective layers around the CNS. Any abnormalities during this developmental phase could potentially predispose individuals to dural defects and the subsequent manifestations of duropathies. Overall, the relationship between dural abnormalities and the spectrum of duropathies underscores the need for further research to elucidate the mechanisms driving these conditions, particularly focusing on embryological factors and their influence on clinical outcomes.

### 4.1. Spontaneous Intracranial Hypotension

SIH is characterized by low CSF volume due to leakage, typically showing low CSF opening pressure (<6 cm of water), although it can also occur with normal pressure [126,128]. The main sources of CSF leaks include tearing of the spinal dura, the rupture of a meningeal diverticulum, and CSF-venous fistulas [129,132]. SIH commonly presents with postural headaches, and additional symptoms may include nausea, neck pain, and dizziness [130]. Diagnosis involves brain MRI, which can reveal stigmata of SIH, alongside assessments of low CSF opening pressure. Given the variability in clinical presentation, a systematic approach to diagnosis and management is essential. Dural tears are associated with several diseases, leading to serious complications such as SS, bibrachial amyotrophy, and spinal cord herniation from persistent spinal CSF leaks [131]. A cohort study showed that 6 out of 51 patients developed SS, with the risk of complications significantly increasing over time. This highlights the importance of early intervention for patients with persistent CSF leaks to mitigate these risks [131].

Most spontaneous CSF leaks occur in the spinal region, especially at the thoracic or cervicothoracic junction, with less frequent occurrences at the skull base and sacral regions [133]. The diagnostic criteria for SIH necessitate objective evidence, which may include MRI findings indicative of SIH (e.g., pachymeningeal enhancement, brain sagging, subdural fluid collections), spinal imaging showing a CSF leak, or low CSF opening pressure [134]. A ventral spinal CSF leak can be diagnosed radiographically if the extrathecal CSF collection is confined to the ventral aspect of the common thecal sac or if a ventral dural tear is identified using advanced imaging techniques like digital subtraction myelography or dynamic CT myelography.

Given the variability in clinical presentations, a systematic approach for diagnosing and managing SIH is crucial for facilitating quicker diagnosis and treatment. Spinal MRI plays a vital role in the diagnostic process, particularly in identifying whether a longitudinal extradural collection is present, which influences the choice of further diagnostic imaging methods.

Dural tears commonly indicate SIH but can also lead to serious long-term complications, including superficial siderosis, bibrachial amyotrophy, and spinal cord herniation, especially in patients with ventral spinal CSF leaks. Approximately 25% of SIH patients present with a ventral spinal CSF leak, which can become chronic and persist for years despite a gradual reduction in headache severity [135]. A cohort study tracking patients with ventral spinal CSF leaks identified that the risk of developing superficial siderosis or bibrachial amyotrophy increases substantially over time, rising from 0% at 48 months to nearly 60% at 192 months. Both conditions can result in significant disability, emphasizing the importance of early intervention to prevent neurological impairments [135].

### 4.2. Multisegmental (or Bibrachial) Amyotrophy

Multisegmental Amyotrophy is characterized by progressive weakness and atrophy of upper extremity muscles, often mimicking amyotrophic lateral sclerosis (ALS) [131,136,137,138]. This condition may result from cervical nerve root stretching over extradural CSF collections. This pathophysiological theory is supported by findings indicating significant enlargement of the extradural CSF collection over time in patients who developed bibrachial amyotrophy. Classic infratentorial SS typically leads to sensorineural hearing loss and cerebellar ataxia, with around 5–10% of cases exhibiting lower motor neuron features [139]. The connection between SIH and SS is well-established, with SIH often preceding SS. The majority of infratentorial SS cases result from a dural defect causing persistent low-volume bleeding into the subarachnoid space due to fragile vessels. Additionally, extra-arachnoid fluid collections from CSF extravasation are frequently documented, predominantly located ventrally and extending longitudinally, leading to the term VLISFC.

Beyond SS, ventral spinal CSF leaks are associated with long-term complications such as bibrachial amyotrophy and spinal cord herniation [136]. Approximately 25% of SIH patients present with a ventral spinal CSF leak, which can become chronic and persist for years despite a gradual reduction in headache severity. Pathological analyses have indicated that VLISFC is critical in anterior horn damage, ruling out hemosiderin deposition as the primary cause [140]. The anterior motor nerve roots, particularly at C5, appear especially vulnerable due to tethering of the cervical cord.

### 4.3. Spontaneous Transdural Spinal Cord Herniation

Spontaneous Transdural Spinal Cord Herniation (STSCH) is a rare condition where the spinal cord protrudes through a dural defect, usually presenting with Brown-Sequard syndrome or spastic paraparesis [141,142,143,144,145,146,147]. The prevailing theory suggests that STSCH results from an initial dural tear followed by progressive herniation. Surgical intervention is crucial, often involving techniques such as dural defect enlargement and duraplasty.

The etiology of STSCH remains debated; however, the predominant theory suggests that it arises from an initial dural tear, followed by gradual herniation of the spinal cord through this tear. Early tamponade of the dural defect by the spinal cord may lead to milder symptoms of SIH, causing patients to seek medical attention less frequently, which could increase the risk for herniation.

The embryological origins of STSCH are complex. During neural tube formation, neural crest cells contribute to the development of the meninges, including the dura mater. The formation of a transdural appendix—an aggregate of non-functional neuronal cells adjacent to the spinal cord—could occur during early gestation, leading to a defect in the dura or PLL (if present) [11,148]. This condition is hypothesized to result from improper fusion of the somites during embryonic development and may lead to tethering of the spinal cord, contributing to neurological deficits.

### 4.4. Spinal Arachnoid Web

Spinal Arachnoid Web (SAW) is an abnormal thickening of arachnoid tissue that may develop post-trauma or be congenital. SAWs are included among duropathies due to their clinical similarities to spinal cord herniation [149,150]. Clinical signs often include sensory disturbances, pain, and weakness, primarily affecting the lower limbs [149,150,151,152,153,154,155,156,157,158,159].

SAW was not initially classified as a “duropathy,” but recent proposals from neurosurgical authors have included it within this category due to its clinical manifestations resembling those of spinal cord herniation. SAW is characterized by an abnormal thickening of the bands of intradural arachnoid tissue that extend from the pial surface of the dorsal aspect of the spinal cord [149]. These webs may be considered variants of arachnoid cysts, remnants of disrupted or collapsed cysts, or incomplete formations of arachnoid cysts [149].

SAWs may develop following a clear history of trauma, which supports the theory of a preceding arachnoid cyst. However, there exists a category of non-traumatic arachnoid webs with unknown etiology, potentially congenital and associated with a thickened ligamentum flavum [153,154].

The pathophysiology of SAWs remains poorly understood, but it is hypothesized that they may arise from segmental inflammation of the arachnoid mater or intermediate leptomeninges, potentially triggered by trauma, surgery, hemorrhage, or infection [154,160,161,162].

### 4.5. Superficial Siderosis

SS is characterized by hemosiderin deposits on the CNS surface and is commonly associated with chronic CSF leaks. Long-term follow-up indicates that many patients may remain asymptomatic despite the presence of superficial siderosis [130,131]. Surgical intervention aims to stop ongoing bleeding and prevent further progression of the condition, emphasizing the need for early intervention.

SS of the CNS is characterized by the deposition of hemosiderin on the brain and spinal cord surfaces. In its classic infratentorial form, these deposits are primarily concentrated in the superior cerebellum, and patients typically do not have a history of intracranial bleeding [130]. The clinical triad associated with infratentorial superficial siderosis includes gradually worsening hearing loss, ataxia, and myelopathy.

Approximately half of the patients with SS have a spinal dural defect, where chronic hemorrhage at the leak site is likely responsible for the development of this condition [130]. A study indicated that long-term follow-up revealed two-thirds of patients with superficial siderosis remained asymptomatic, with MRI findings suggesting a limited burden of siderosis. However, it remains uncertain whether these patients would have developed symptoms if the spinal CSF leak had gone untreated. Serial imaging often displayed progressive superficial siderosis, indicating ongoing subarachnoid bleeding until surgical repair of the CSF leak was performed. Dural tears can also be linked to protruding disks, osteophytes, or a combination of these factors, as well as calcifications between the dura and arachnoid. In some cases, a herniated thoracic disk without an apparent dural defect has been suspected to cause superficial siderosis in patients with intraspinal fluid collections.

SS is typically a slowly progressive disorder. If a source of bleeding is identified and surgically addressed, some patients may experience clinical improvement. The primary goal of surgical intervention is to prevent further progression by removing the source of chronic bleeding. However, predicting which patients will benefit from surgery can be challenging. In cases of long-standing superficial siderosis, the benefits of removing the bleeding source may be less clear, and concerns arise regarding continued disease progression despite surgical correction. Chronic superficial siderosis often results in irreversible neural tissue damage, limiting the potential benefits of any intervention. Hemosiderin-induced neural tissue damage is likely progressive, and some authors suggest that continued progression despite therapy may relate to a neurodegenerative cascade initiated by long-standing siderosis, leading to the failure of reparative mechanisms after reaching a “point of no return”.

## 5. Conclusions

In conclusion, the term “duropathies” encapsulates a range of conditions linked by common etiology, primarily involving spinal dural tears and CSF leaks. These abnormalities, including SIH, SS, multisegmental amyotrophy, and spinal cord herniation, demonstrate overlapping clinical presentations and significant interrelationships. The complexities of these conditions underscore the necessity for a thorough understanding of their pathophysiology to inform effective diagnostic and therapeutic strategies.

SIH is particularly significant, as it leads to a variety of neurological symptoms and highlights the critical role of the meninges in CSF homeostasis. The presence of dural tears can lead to serious complications like SS and spinal cord herniation, emphasizing the importance of early diagnosis and intervention. The review of spinal arachnoid webs and their potential clinical implications further illustrates the breadth of duropathies.

Ultimately, a multidisciplinary approach involving neurology, neurosurgery, and imaging specialists is essential for optimal management of these conditions. Regular monitoring and prompt treatment can mitigate long-term neurological consequences, improving patient outcomes and quality of life.

## Figures and Tables

**Table 1:** Regulatory pathways of meningeal development.

Issues	Features
Neural Crest Contributions	Neural crest-derived cells from the caudal forebrain and midbrain contribute to the forebrain meninges; mesoderm-derived cells form the midbrain and hindbrain meninges [19,25,26].
Endothelial Cell Origin	Endothelial cells in all meningeal regions are of mesoderm origin [19].
Histological Findings	Cranial meninges originate from both neural crest (ectoderm) and mesoderm, specifically the prechordal plate and paraxial mesoderm [11].
Lineage Tracing in Mice	Cre-loxP technology allows for the tracing of specific cell populations [27]. Studies show neural crest cells populate forebrain meninges but not midbrain or hindbrain [28].
Mesp1-Cre Findings	Mesp1-Cre indicates midbrain and hindbrain meninges are of mesoderm origin, while forebrain meninges come from neural crest [13].
Molecular Regulation	Poorly understood.
Foxc1 Gene	Key transcription factor in meningeal development; expressed throughout the primary meninx [29,30]. Linked to congenital hydrocephalus via a mutation, causing severe meningeal defects [31].
Foxc1 Mutants	Show compact meningeal mesenchyme and loss of arachnoid and dura mater differentiation [14].
Zic Family Genes	Zic1 and Zic3 expressed in meninges; double mutation reduces meningeal fibroblast proliferation and disrupts pial basement membrane [32].
Twist1 Gene	A transcription factor involved in meningeal development; deletion causes hypoplasia of dura and arachnoid mater [33].
TGFβ Signaling	Required for normal meningeal development; Tgfbr2 deletion results in failure of forebrain meningeal development [34].
WNT/β-Catenin Signaling	Promotes cell proliferation in meningeal layer; excessive RA leads to thin meninges [28,35].
Gene Disruptions	ECM or cell adhesion genes (e.g., Apbb1, Col4a1) disrupt pial basement membrane; essential for structural integrity rather than developmental regulation [36,37].

**Table 2:** Relations between meningeal development and calvarium.

Issues	Features
Calvaria Composition	Five bones: a pair of frontal bones, a pair of parietal bones, and an interparietal bone, joined by soft connective tissue (sutural tissue) [38,39].
Development Origin	It develops from the mesenchyme that envelops the brain, with progenitor cells present in the primary meninx. The calvaria has a dual origin: the frontal bone primarily arises from neural crest cells, while the parietal bone is entirely derived from mesodermal cells [13,40].
Dura Mater Role	The outer layer of the dura mater acts as the periosteum on the inner surface of the calvarial bone. Surgical interventions have demonstrated that the dura mater is essential for re-ossification and for maintaining the patency of cranial sutures [40,41,42,43].
Molecular Interactions	Various secreted molecules, such as TGFβ, FGF, and BMP, are expressed in the dura mater and play a role in mediating interactions with the calvaria [44,45,46,47,48].
Early Development Interactions	Early interactions between the meninges and the calvaria are not as extensively studied; however, evidence suggests a correlation between normal development and mutant phenotypes. The dural limiting layer is observed to appear at stage 17 in humans [14,19].
Calvarial Growth Correlation	Frontal and parietal bones arise from mesenchyme on the basolateral side of the brain and expand apically around ~E12.5 in mice, correlating with the dural limiting layer [38,39,49,50].
Foxc1 Mutants Findings	Foxc1 mutants (Foxc1ch/ch and Foxc1lacZ/lacZ) exhibit severe meningeal defects and absent calvarial bone on the apical side at birth. Calvarial development is arrested at E13.5, indicating Foxc1’s role in calvarial growth [29,51].
Tgfbr2 Knockout Findings	Neural crest-specific Tgfbr2 knockout mutants exhibit significant meningeal and calvarial defects, highlighting that Tgfbr2 regulates the development of the parietal bone in a cell non-autonomous manner [34].
Retinoic Acid (RA) Treatment	Treatment with RA in mouse embryos resulted in meningeal defects and a partial to complete loss of parietal and interparietal bones at E17.5 [28]. RA is known to upregulate anti-osteogenic genes, which inhibit osteogenic specification in calvarial mesenchyme [38].
Cyp26b1 Enzyme Role	Calvarial mesenchyme expresses RA-degrading enzyme Cyp26b1 at E14.5, and inactivation leads to severe hypoplasia of calvarial bone [52,53].

**Table 3:** Spinal dural arteries [101].

Type of Artery	Quantity per Spinal Nerve	Description
Short Arteries	5 to 10	Supply the dural sheath of the corresponding nerve root.
Medium Arteries	2 or 3	Follow either the cranial or caudal aspect of the nerve root sheath, branching into a vertical branch (upward or downward) or two transverse branches (ventral and dorsal), providing blood to the dural sheath of the nerve roots and the adjacent spinal dura mater.
Long Arteries	1 or 2	Travel medially along the spinal nerve sheath and bifurcate into terminal branches that irrigate the spinal dura mater, including vertical (cranial and caudal), transverse (dorsal), ventrocranial, and ventrocaudal branches.

## Data Availability

No new data were created or analyzed in this study.
